# Effects of Acupuncture on Cerebral Blood Flow in Episodic Migraine Patients Investigated by Arterial Spin Labeling MRI

**DOI:** 10.1155/np/3493770

**Published:** 2026-10-06

**Authors:** Tomokazu Kikuchi, Satoru Yamaguchi, Hiroshi Matsuda, Kazuhiro Tsuchiya, Nobuo Araki

**Affiliations:** ^1^ Department of Oriental Medicine Saitama Medical University Saitama Japan saitama-med.ac.jp; ^2^ The Japan School of Acupuncture, Moxibustion and Physiotherapy Tokyo Japan jsam.jp; ^3^ Clinical Imaging Center for Healthcare Nippon Medical School Tokyo Japan nms.ac.jp; ^4^ Integrative Brain Imaging Center National Center of Neurology and Psychiatry Tokyo Japan ncnp.go.jp; ^5^ Department of Radiology Saitama Medical Center Saitama Medical University Saitama Japan saitama-med.ac.jp; ^6^ Department of Neurology Saitama Medical School Saitama Japan

## Abstract

**Objective:**

To investigate the temporal dynamics of cerebral blood flow (CBF) responses to Japanese‐style acupuncture in patients with episodic migraine using arterial spin labeling (ASL) magnetic resonance imaging (MRI) and to compare these responses with those of healthy controls.

**Methods:**

Patients with episodic migraine were recruited according to the International Classification of Headache Disorders, 3rd edition (ICHD‐3), and healthy volunteers without migraine served as controls. Japanese‐style acupuncture was administered for 10 min at four clinically relevant cranio‐cervical acupoints (ST8, ST6, GB21, and GB12) using nonmagnetic silver needles (0.2 mm in diameter). All acupuncture procedures were performed by the same licensed acupuncturist. CBF was assessed during the interictal period using repeated ASL MRI at baseline, during acupuncture stimulation (early and late phases), immediately after needle removal, and 15 and 30 min after stimulation.

**Results:**

Eleven patients with episodic migraine (3 men and 8 women; mean age, 39.1 ± 4.6 years) and 11 healthy volunteers (4 men and 7 women; mean age, 32.3 ± 9.2 years) were included in the final analysis. Both groups demonstrated increased cerebral perfusion within the operculum, insula, cingulate cortex, and thalamus during acupuncture stimulation. In healthy controls, these perfusion changes gradually returned toward baseline following stimulation. In contrast, patients with episodic migraine exhibited more prolonged cerebral perfusion responses that persisted after needle removal and extended into frontal cortical regions. Compared with healthy controls, migraine patients demonstrated greater temporal persistence of cerebral perfusion modulation.

**Conclusions:**

Temporally organized cerebral perfusion changes were observed during and after Japanese‐style acupuncture in both healthy controls and patients with episodic migraine. Compared with healthy controls, patients with episodic migraine exhibited cerebral perfusion changes that remained evident during the poststimulation period. These exploratory findings suggest differences in the temporal dynamics of cerebral perfusion between the two groups. Further controlled studies are required to determine the specificity and physiological significance of these responses.

**Trial Registration** : UMIN Clinical Trials Registry (UMIN‐CTR): UMIN000005644

## 1. Introduction

Migraine is a common neurological disorder characterized by recurrent headache attacks and associated symptoms such as nausea, photophobia, and phonophobia. It represents a major cause of disability worldwide and contributes substantially to the global burden of neurological disease [1]. Although pharmacological therapies, including calcitonin gene‐related peptide (CGRP)‐targeted agents, have substantially improved migraine management, a considerable proportion of patients either fail to achieve adequate symptom control or seek effective nonpharmacological therapeutic options [2–4].

Acupuncture has been used for the treatment of headache disorders for more than 2000 years and is recommended as a therapeutic option for migraine in the Japanese Clinical Practice Guideline for Chronic Headache, 2021 [4]. Clinical trials and systematic reviews have demonstrated that acupuncture reduces headache frequency, alleviates headache‐related disability, and improves quality of life in patients with migraine [5–8]. Despite accumulating clinical evidence, the central neurophysiological mechanisms underlying acupuncture remain incompletely understood.

Recent neuroimaging studies have demonstrated that migraine is associated with abnormal activity and connectivity within distributed pain‐related brain networks, including the anterior cingulate cortex (ACC), insula, thalamus, medial prefrontal cortex (mPFC), and frontoparietal network [9, 10]. These abnormalities persist even during the interictal period, suggesting that migraine is characterized by altered neurovascular regulation and dysfunctional brain network organization rather than episodic headache attacks alone. Functional magnetic resonance imaging (fMRI) and arterial spin labeling (ASL) MRI have therefore become valuable tools for investigating the central mechanisms of migraine and evaluating physiological responses to therapeutic interventions [11–16].

Most neuroimaging studies investigating acupuncture for migraine have evaluated brain responses before and after treatment or have employed standardized traditional Chinese medicine (TCM) protocols using distal acupoints selected according to the meridian theory [8, 11, 17]. In contrast, Japanese‐style acupuncture emphasizes individualized treatment based on palpatory findings and commonly applies gentle stimulation to clinically relevant cranio‐cervical regions associated with migraine symptoms. Furthermore, little is known about how cerebral perfusion evolves over time during and after acupuncture stimulation, particularly when Japanese‐style acupuncture is applied. This represents an important gap in the current understanding of the central mechanisms of acupuncture.

ASL MRI is a noninvasive imaging technique that enables assessment of cerebral perfusion without the use of exogenous contrast agents [18]. Because repeated ASL acquisitions can be performed during a single imaging session, this technique is particularly well suited for evaluating the temporal evolution of cerebral perfusion during and after acupuncture stimulation. Previous ASL studies have demonstrated that cerebral perfusion in migraine varies according to the phase of the migraine cycle and clinical subtype. Meylakh et al. [19] reported reduced regional cerebral blood flow (CBF) in the lateral hypothalamus and visual cortex immediately before migraine headache, whereas Stankewitz et al. [20] demonstrated dynamic perfusion changes across the migraine cycle involving limbic and pain‐processing regions. Interictal studies have also identified altered regional perfusion in patients with migraine without aura, including frontal and visual processing regions. Furthermore, differences in cerebral perfusion between migraine with and without aura have been reported in several regions, including the thalamus and occipital cortex. Collectively, these findings indicate that ASL can detect state‐ and subtype‐dependent cerebral perfusion patterns in migraine, although their temporal characteristics and responses during sensory interventions remain incompletely understood.

Previous ASL studies indicate that occipital perfusion abnormalities may be present in migraine independently of experimental stimulation. In particular, Fu et al. [21] reported interictal differences in cerebral perfusion between patients with migraine with and without aura, including lower perfusion in the medioventral occipital cortex and thalamus in patients with aura. Zhang et al. [22] also demonstrated altered interictal regional perfusion and perfusion connectivity in patients with episodic migraine without aura. Therefore, the relative occipital perfusion pattern observed in the present study may partly reflect pre‐existing migraine‐related or subtype‐related cerebral perfusion characteristics rather than an effect associated exclusively with acupuncture. This interpretation is particularly relevant because our migraine group included both patients with and without aura, and the small sample size did not permit meaningful subtype‐specific analysis.

Therefore, the present study investigated the temporal dynamics of CBF responses to Japanese‐style acupuncture in patients with episodic migraine and healthy controls using repeated ASL MRI. We hypothesized that patients with episodic migraine would exhibit greater and more prolonged cerebral perfusion responses than healthy controls, reflecting the altered neurovascular regulation associated with migraine.

## 2. Materials and Methods

### 2.1. Participants

Patients with episodic migraine were recruited according to the International Classification of Headache Disorders, 3rd edition (ICHD‐3) [4]. Both migraine with aura and migraine without aura were eligible for inclusion. Healthy volunteers without a history of primary headache disorders were recruited as controls.

The main exclusion criteria included cerebrovascular disease, diabetes mellitus, hypertension, cardiovascular disease, chronic pain conditions other than headache, smoking, insomnia, current use of preventive migraine medication, triptan use within 24 h before the MRI examination, and chronic migraine.

All MRI examinations were performed during the interictal period. Written informed consent was obtained from all participants prior to enrollment. The study protocol was approved by the appropriate institutional ethics committee and was conducted in accordance with the Declaration of Helsinki.

### 2.2. Acupuncture Procedure

Japanese‐style acupuncture was administered by a licensed acupuncturist with extensive clinical experience in headache treatment. Point selection followed routine Japanese clinical practice and was guided by palpatory findings rather than a standardized protocol based solely on meridian theory.

Acupuncture stimulation was delivered using nonmagnetic silver needles (0.2 × 50 mm; Aoki Shin‐i Co., Japan) inserted bilaterally at four clinically relevant cranio‐cervical acupoints: ST8 (temporalis muscle), ST6 (masseter muscle), GB21 (upper trapezius muscle), and GB12 (splenius capitis muscle). These acupoints are frequently used in Japanese clinical practice for migraine and are anatomically related to trigeminal and upper cervical afferent pathways implicated in headache pathophysiology [5, 10, 11, 17].

Needles were inserted under sterile conditions and retained for 10 min without manual manipulation, electrical stimulation, or intentional elicitation of the deqi sensation. All acupuncture procedures were performed within the MRI suite under standardized environmental conditions to minimize variability during image acquisition.

### 2.3. MRI Acquisition

Cerebral perfusion images were acquired using a 3‐Tesla MRI scanner (MAGNETOM Verio, Siemens Healthineers, Erlangen, Germany) with a pulsed ASL (PASL) sequence (ep2d_pasl) [23]. Six whole‐brain ASL scans were serially obtained for each participant using the QUIPSS II technique with thin‐slice TI1 periodic saturation. In this PASL protocol, TI1, corresponding to the bolus duration, was 700 ms, and TI2, corresponding to the inversion time between the labeling and image acquisition, was ~1800 ms.

Six ASL scans were acquired in a fixed temporal sequence: one at baseline, two during acupuncture stimulation, and three after needle removal, including immediately after stimulation and at 15 and 30 min after stimulation. Acquisition of each ASL scan duration: ~4 min. The participants rested with their eyes closed and were instructed to remain awake throughout the examination.

All ASL images were spatially normalized to an a priori gray matter template in Montreal Neurological Institute (MNI) standard space using Statistical Parametric Mapping 8 (SPM8). The images were subsequently smoothed using a 12‐mm full width at half‐maximum Gaussian kernel. Proportional scaling was performed in SPM8, with the global mean cerebral perfusion scaled to 50 mL/100 g/min. Accordingly, the scaled voxel‐wise maps were used to evaluate relative regional perfusion patterns and were not interpreted as measures of absolute CBF or true global perfusion changes.

### 2.4. CBF Quantification

CBF maps were calculated from the difference between the label and control images according to the standard kinetic model for PASL. For PASL with QUIPSS II, the calculation incorporated the blood–brain partition coefficient (*λ* = 0.9 mL/g), labeling efficiency (*α* = 0.95), equilibrium magnetization of the brain tissue (M0), TI1 (700 ms), TI2 (~1800 ms), and the longitudinal relaxation time of arterial blood (T1b = 1664 ms). Conversion to CBF units of mL/100 g/min was performed within the analysis software.

Because proportional scaling to a global mean of 50 mL/100 g/min was subsequently applied in SPM8, the resulting voxel‐wise maps were used to evaluate relative regional perfusion patterns rather than absolute global CBF changes. After spatial normalization, CBF maps were generated for each time point and subsequently subjected to proportional scaling, as described above.

### 2.5. Statistical Analysis

Statistical analyses were performed using SPM8. We employed a two‐sample *t*‐test for comparison of baseline perfusion between migraine and normal control groups. Then, we employed a flexible factorial design to evaluate the main effects of time (baseline, stim, and post) and the group × time interaction. In the interaction analysis, we specifically examined the differences in (stim − rest) and (post − rest) contrasts between the migraine and normal control groups. The set of values resulting from the SPM8 analysis constituted a statistical parametric map of the *t*‐statistic, SPM{*t*}. The SPM{*t*} maps were then transformed into units of normal distribution (SPM{*Z*}). The height threshold was set to uncorrected *p*  < 0.01 for the whole‐brain analysis. Although a more stringent threshold (e.g., family‐wise error [FWE] correction) is generally preferred to ensure high reliability, we prioritized the identification of potential neural response patterns in this exploratory study with a limited sample size. To mitigate the risk of false positives, we combined this voxel‐wise threshold with a cluster‐size threshold, reporting only those clusters that exceeded 100 voxels. The results are presented using *Z*‐scores, which are standard for normalizing statistical values in SPM analysis. The coordinates of the significant clusters are reported in Talairach space [24] using the mni2tal script [25].

### 2.6. Ethical Approval

This study was approved by the Research Ethics Committee of Saitama Medical University. Approval Number 431, June 2011. Participants were provided with written and verbal explanations about the freedom of study participation/withdrawal, consent, and protection of privacy. They then provided written consent. All study procedures were performed in accordance with the principles of the Declaration of Helsinki.

## 3. Results

### 3.1. Participant Flow

Twenty‐five participants were initially enrolled. One patient with episodic migraine and two healthy controls were excluded because of insufficient image quality. Consequently, 11 patients with episodic migraine and 11 healthy controls were included in the final analysis (Figure 1).

**Figure 1:**
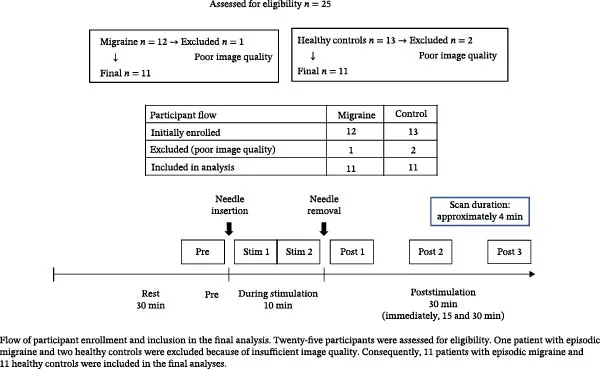
Study design and experimental timeline.

### 3.2. Participant Characteristics

The migraine group consisted of 3 men and 8 women with a mean age of 39.1 ± 4.6 years. Three patients had migraine with aura, and eight had migraine without aura. No participant was receiving preventive migraine medication at the time of the study. The healthy control group consisted of 4 men and 7 women with a mean age of 32.3 ± 9.2 years. Detailed demographic and clinical characteristics are summarized in Table 1.

**Table 1:** Demographic and clinical characteristics of the study participants.

Characteristic	Episodic migraine (*n* = 11)	Healthy controls (*n* = 11)
Age (years), mean ± SD	39.1 ± 4.6	32.3 ± 9.2
Female, *n* (%)	8 (72.7)	7 (63.6)
Male, *n* (%)	3 (27.3)	4 (36.4)
Migraine with aura, *n* (%)	3 (27.3)	—
Migraine without aura, *n* (%)	8 (72.7)	—
Age at onset (years), mean ± SD	20.0 ± 6.1^a^	—
Disease duration (years), mean ± SD	19.1 ± 7.9	—
Monthly headache days, median (range)	4 (2–5)	—
Preventive migraine medication, *n* (%)	0 (0)	—
Acute medication use, *n* (%)	11 (100)	—
└ Triptans	10 (90.9)	—
└ NSAIDs	1 (9.1)	—

*Note:* baseline demographic and clinical characteristics of patients with episodic migraine and healthy controls included in the final analysis. Continuous variables are presented as mean ± standard deviation (SD) or median (range), as appropriate. Categorical variables are presented as number (%).

Abbreviation: NSAIDs, nonsteroidal anti‐inflammatory drugs.

^a^Age at onset was self‐reported, and disease duration was calculated by subtracting the self‐reported age at migraine onset from age at the time of the study.

### 3.3. Baseline Cerebral Perfusion

Before acupuncture stimulation, patients with episodic migraine demonstrated higher cerebral perfusion within the right occipital and temporal cortices and lower perfusion within the left temporoparietal cortex and cingulate gyrus compared with that in healthy controls (Figure 2).

**Figure 2:**
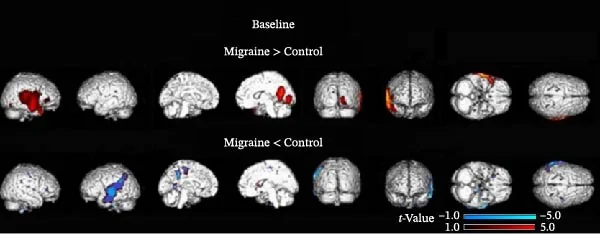
Comparison of baseline cerebral perfusion between migraine and control groups. Voxel‐wise comparison of baseline cerebral blood flow between patients with episodic migraine and healthy controls before acupuncture stimulation, assessed using arterial spin labeling (ASL) MRI.

### 3.4. Temporal Cerebral Perfusion Responses

During acupuncture stimulation, both groups demonstrated increased cerebral perfusion within the operculum, insula, cingulate cortex, and thalamus. However, the magnitude and temporal characteristics of these perfusion responses differed between groups (Figures 3 and 4).

**Figure 3:**
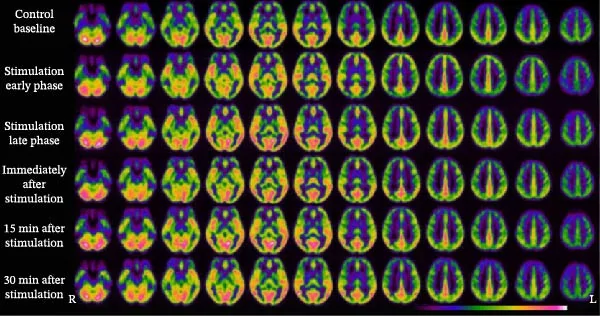
Temporal cerebral perfusion responses in the control group. Voxel‐wise analysis showing brain regions with significantly increased and decreased cerebral perfusion relative to baseline during and after Japanese‐style acupuncture in healthy controls.

**Figure 4:**
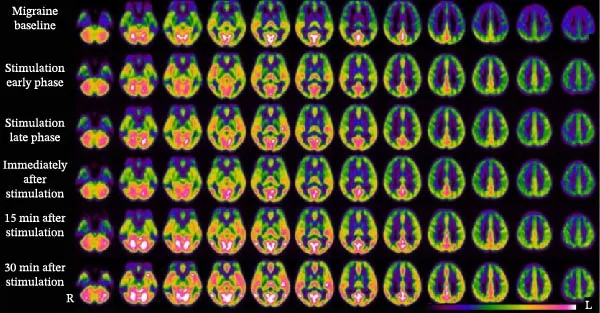
Temporal cerebral perfusion maps in the migraine group. Arterial spin labeling (ASL) MRI perfusion maps are shown at baseline, during the early and late phases of acupuncture stimulation, and immediately, 15 min, and 30 min after stimulation in patients with episodic migraine.

In healthy controls, cerebral perfusion increased during acupuncture but gradually returned toward baseline immediately after needle removal (Figure 5). In contrast, patients with episodic migraine exhibited persistent cerebral perfusion responses after stimulation, with increased perfusion remaining evident immediately after needle removal and at both 15 and 30 min after stimulation. These prolonged responses extended to frontal cortical regions (Figure 6).

**Figure 5:**
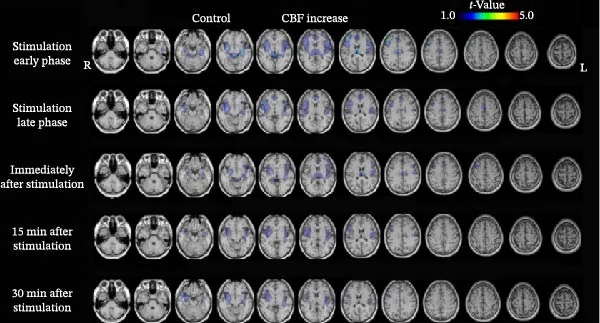
Cerebral blood flow (CBF) increases during and after acupuncture stimulation in the control group. Voxel‐wise statistical maps showing increased cerebral perfusion relative to baseline during the early and late phases of stimulation and immediately, 15 min, and 30 min after stimulation in healthy controls.

**Figure 6:**
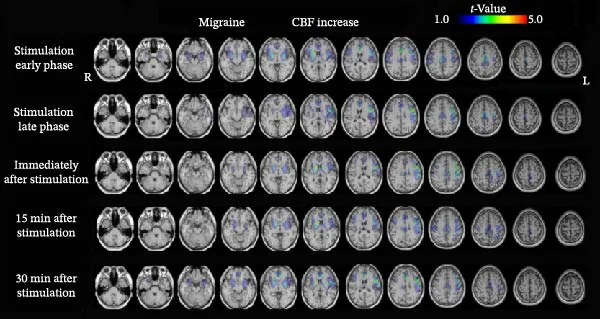
Cerebral blood flow (CBF) increases during and after acupuncture stimulation in the migraine group. Voxel‐wise statistical maps showing increased cerebral perfusion relative to baseline during the early and late phases of stimulation and immediately, 15 min, and 30 min after stimulation in patients with episodic migraine.

In addition, the scaled perfusion maps showed relatively lower occipital perfusion during and after acupuncture in patients with episodic migraine, whereas no comparable change was observed in healthy controls (Figure 7).

**Figure 7:**
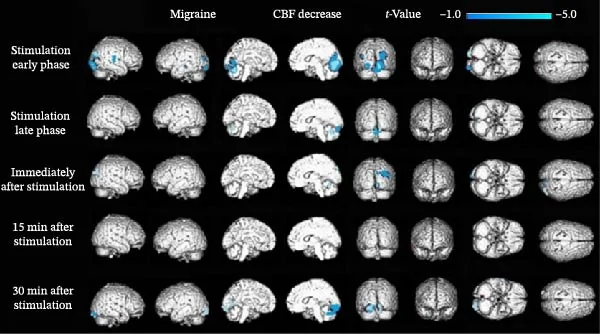
Relative cerebral blood flow (CBF) decreases during and after acupuncture stimulation in the migraine group. Voxel‐wise statistical maps showing lower cerebral perfusion relative to baseline during the early and late phases of stimulation and immediately, 15 min, and 30 min after stimulation in patients with episodic migraine. These maps reflect relative perfusion differences following proportional scaling and do not establish an absolute decrease in CBF.

The interaction between the group and experimental condition for opercular perfusion is presented in Figure 8 and Table 2.

**Figure 8:**
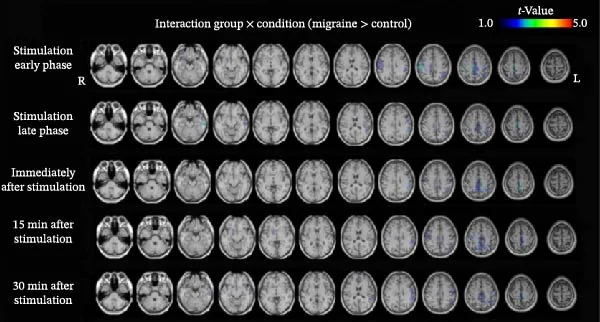
Group × condition interaction in cerebral blood flow (CBF) changes (migraine > control). Voxel‐wise statistical maps showing between‐group differences in changes from baseline during the early and late phases of acupuncture stimulation and immediately, 15 min, and 30 min after stimulation. The displayed contrast is migraine > control.

**Table 2:** Peak coordinates for the group × condition interaction (migraine > control).

Time point	Talairach coordinate			*Z*‐score	Location		
	*X*	*Y*	*Z*				
Stimulation early phase	16	−39	44	3.78	Right	Cingulate gyrus	BA31
	41	−20	51	3.6	Right	Postcentral gyrus	BA3
	−10	−19	49	3.13	Left	Medial frontal gyrus	BA6
Stimulation late phase	−12	−19	47	3.21	Left	Medial frontal gyrus	BA6
	12	−21	49	3.09	Right	Medial frontal gyrus	BA6
	−60	−19	−16	3.06	Left	Inferior temporal gyrus	BA20
	−45	−38	28	2.86	Left	Supramarginal gyrus	BA40
Immediately after stimulation	−12	−19	47	3.8	Left	Medial frontal gyrus	BA6
	12	−16	41	2.96	Right	Cingulate gyrus	BA24
	−53	−38	33	2.95	Left	Supramarginal gyrus	BA40
15 min after stimulation	−16	−55	47	3.14	Left	Precuneus	BA7
	14	−44	49	3.07	Right	Precuneus	BA7
	48	−17	48	2.78	Right	Postcentral gyrus	BA3
30 min after stimulation	−10	−19	49	2.72	Left	Medial frontal gyrus	BA6
	−51	−36	35	2.65	Left	Inferior parietal lobule	BA40

*Note:* peak brain regions identified by voxel‐wise statistical analyses during and after Japanese‐style acupuncture. Coordinates are reported in Talairach space and statistical values are presented as *Z*‐scores.

Abbreviation: BA, Brodmann area.

## 4. Discussion

The apparent reduction in occipital perfusion should be interpreted cautiously because proportional scaling was applied to all perfusion maps. Scaling each image to a fixed global mean removes information regarding the absolute global CBF and expresses regional values relative to the global perfusion level. Consequently, an apparent decrease in one region may partly reflect relative increases in other regions rather than an absolute reduction in local tissue perfusion. In particular, the lower relative occipital perfusion observed in patients with episodic migraine may have been influenced by concurrent relative increases in the frontal, insular, or other regions. Therefore, the present data do not establish an absolute decrease in occipital CBF.

### 4.1. Principal Findings

The present study identified temporally distinct cerebral perfusion patterns during and after Japanese‐style acupuncture in both healthy controls and patients with episodic migraine. Although both groups exhibited increased perfusion within pain‐related cortical regions during acupuncture stimulation, the temporal profile differed markedly between the groups. In healthy controls, perfusion changes were largely confined to the stimulation period and gradually returned toward baseline after needle removal. In contrast, patients with episodic migraine demonstrated persistent cerebral perfusion responses extending into the poststimulation period with additional recruitment of frontal cortical regions.

An important strength of the present study is the repeated acquisition of ASL MRI before, during, and after acupuncture stimulation. Unlike conventional neuroimaging studies that compare only pre‐ and posttreatment scans, the present protocol enabled the visualization of the temporal evolution of cerebral perfusion throughout stimulation and the subsequent recovery period. To our knowledge, few previous studies have characterized these dynamic perfusion responses using serial ASL measurements in patients with episodic migraine. The present findings therefore provide exploratory information regarding the temporal organization of cerebral perfusion patterns observed during and after Japanese‐style acupuncture.

Because all examinations were performed during the interictal period, the observed perfusion patterns are unlikely to reflect acute ictal phenomena. They may instead be related to differences in neurovascular regulation or responsiveness of pain‐related brain networks that persist between migraine attacks. However, because the study did not include a sham acupuncture or rest‐only control condition and the serial scans were acquired in a fixed temporal order, the poststimulation perfusion patterns cannot be attributed specifically to acupuncture.

### 4.2. Characteristics of Japanese‐Style Acupuncture

Most previous neuroimaging studies investigating acupuncture for migraine have adopted TCM protocols using standardized distal acupoints, such as LI4 and ST36, selected according to meridian theory [8, 11, 17]. In contrast, the present study investigated Japanese‐style acupuncture, which emphasizes individualized treatment based on palpatory findings and clinical assessment rather than standardized point prescriptions.

In the present study, acupuncture was applied to cranio‐cervical regions (ST8, ST6, GB21, and GB12) that are frequently involved in migraine symptoms and are anatomically related to trigeminal and upper cervical afferent pathways implicated in migraine pathophysiology [5, 10]. However, in the absence of a sham acupuncture or other sensory control condition, the observed cerebral perfusion responses cannot be distinguished from responses to nonspecific somatosensory stimulation.

Another characteristic of Japanese acupuncture is the use of relatively fine needles and gentle stimulation. Whereas many TCM‐based neuroimaging studies employ thicker needles and intentionally elicit pronounced *deqi* sensations [5, 10], the present study used 0.2‐mm silver needles without manual manipulation or electrical stimulation. Nevertheless, cerebral perfusion changes were observed during and after this relatively gentle stimulation protocol. These findings indicate that measurable cerebral perfusion responses can occur even in the absence of intense peripheral stimulation or intentional elicitation of strong *deqi* sensations. However, the physiological specificity of these responses remains to be established in appropriately controlled studies.

Rather than suggesting the superiority of one acupuncture style over another, the present findings indicate that different acupuncture approaches may engage the central nervous system through distinct patterns of afferent input. Japanese‐style acupuncture, characterized by palpation‐guided point selection and relatively mild cranio‐cervical stimulation, may therefore provide a useful experimental paradigm for investigating cerebral responses to sensory stimulation in patients with migraine using functional neuroimaging.

### 4.3. Temporal Characteristics of Cerebral Perfusion Responses

One of the principal contributions of the present study is the characterization of cerebral perfusion as a dynamic physiological process rather than a static imaging endpoint. Repeated ASL acquisition demonstrated that cerebral perfusion responses evolved sequentially throughout acupuncture stimulation and the poststimulation period.

During the early phase of stimulation, perfusion increases were predominantly observed within the operculum, insula, and primary somatosensory cortex, regions responsible for processing somatosensory afferent input. As stimulation continued, perfusion changes extended toward the ACC and mPFC, regions involved in cognitive pain modulation, emotional processing, endogenous analgesia, and autonomic regulation. Following needle removal, migraine patients continued to demonstrate persistent perfusion changes involving frontal and medial cortical regions, whereas cerebral perfusion in healthy controls gradually returned toward baseline.

The observation that cerebral perfusion changes remained evident during the poststimulation scans suggests that the temporal profile differed between patients with episodic migraine and healthy controls during the experimental session. However, because the scans were acquired in a fixed order without a time‐matched rest‐only condition, these poststimulation changes cannot be distinguished from potential time‐dependent effects. Therefore, they should be regarded as exploratory temporal perfusion patterns rather than definitive evidence of sustained acupuncture‐induced neuromodulation.

The poststimulation perfusion patterns observed in patients with episodic migraine differed from those observed in healthy controls, suggesting possible group differences in cerebral responsiveness during the experimental session. These exploratory findings provide information not only about the spatial distribution of cerebral perfusion patterns but also about their temporal evolution across repeated ASL measurements.

### 4.4. Comparison With Previous Neuroimaging Studies

The present findings are broadly consistent with previous neuroimaging studies demonstrating abnormal functional organization within pain‐related brain networks in migraine. fMRI and ASL studies have repeatedly reported altered activity and connectivity involving the insula, ACC, thalamus, mPFC, and frontoparietal network during both ictal and interictal periods [9, 10, 12, 15, 26]. In particular, recent ASL studies have demonstrated persistent cortical hyperperfusion during the interictal period, suggesting that abnormal neurovascular regulation remains present even between migraine attacks [15].

Previous ASL studies also indicate that occipital perfusion abnormalities may be present in migraine independently of experimental stimulation. Fu et al. [21] reported interictal differences in cerebral perfusion between patients with migraine with and without aura, including lower perfusion in the medioventral occipital cortex and thalamus in patients with aura. Zhang et al. [22] also demonstrated altered interictal regional perfusion and perfusion connectivity in patients with episodic migraine without aura. Therefore, the relative occipital perfusion pattern observed in the present study may partly reflect pre‐existing migraine‐related or subtype‐related cerebral perfusion characteristics rather than a response associated exclusively with acupuncture. This possibility is particularly relevant because our migraine group included both patients with and without aura, and the small sample size did not permit meaningful subtype‐specific analysis.

Our findings extend these previous observations by characterizing cerebral perfusion across multiple time points during and after acupuncture. The serial ASL measurements revealed temporally organized perfusion patterns that differed between patients with episodic migraine and healthy controls during the experimental session. This temporal perspective complements previous cross‐sectional neuroimaging studies and provides exploratory information regarding the dynamic characteristics of cerebral perfusion in migraine.

The progressive transition of perfusion responses from primary somatosensory regions toward medial frontal and cingulate cortices observed in the present study is also consistent with previous fMRI investigations demonstrating that acupuncture influences large‐scale brain networks involved in pain processing, cognitive control, and the default mode network (DMN) [11, 16, 27]. Whereas previous studies primarily evaluated changes in functional connectivity, the present study provides complementary information by characterizing temporal cerebral perfusion patterns using repeated ASL MRI.

Experimental studies have demonstrated that electroacupuncture suppresses CGRP expression within the trigeminovascular system and attenuates trigeminal sensitization in experimental migraine models [28, 29]. Although CGRP concentrations were not measured in the present study, these experimental findings provide a potential biological context for interpreting the cerebral perfusion patterns observed during and after acupuncture. Whether trigeminovascular or CGRP‐related mechanisms contributed to the observed perfusion patterns cannot be determined from the present data.

Clinical studies have further reported that acupuncture may influence autonomic regulation, including heart rate variability (HRV), in patients with migraine [30, 31]. Because cerebral perfusion is influenced by central autonomic networks involving the ACC, mPFC, hypothalamus, and brainstem, autonomic regulation represents one possible context for interpreting the observed perfusion patterns. However, autonomic function was not measured in the present study, and no direct relationship between the observed cerebral perfusion patterns and autonomic activity can be established. Future multimodal studies combining ASL MRI with physiological autonomic measurements will be necessary to investigate this possibility.

Taken together, the observed cerebral perfusion patterns involved regions associated with somatosensory processing, pain regulation, and autonomic function. However, the present ASL data alone cannot determine the underlying neural, trigeminovascular, or autonomic mechanisms. These findings should therefore be regarded as hypothesis‐generating and may help guide future controlled multimodal studies.

### 4.5. Interpretation of Short‐Term Cerebral Perfusion Changes

The present study demonstrated temporal changes in cerebral perfusion during and after acupuncture stimulation. These changes remained evident during the poststimulation scans in patients with episodic migraine, whereas the temporal pattern differed in healthy controls. However, these short‐term perfusion changes should not be interpreted as evidence of neural or functional plasticity.

ASL MRI measures cerebral perfusion and does not directly assess synaptic, structural, or other forms of neuroplasticity. Furthermore, because the serial scans were acquired in a fixed temporal order and no sham acupuncture or rest‐only control condition was included, the observed temporal changes cannot be attributed exclusively to acupuncture stimulation. The present findings should therefore be interpreted as exploratory evidence of differences in cerebral perfusion dynamics during the experimental session.

Future studies combining appropriately controlled longitudinal designs with multimodal measures of neural function will be necessary to determine whether acupuncture‐related cerebral responses are associated with longer‐term changes in brain function.

### 4.6. Limitations

Several limitations of the present study should be acknowledged.

First, this was a pilot study with a relatively small sample size, and no formal sample size calculation was performed. Consequently, the present findings should be regarded as exploratory and require confirmation in larger independent cohorts.

Second, no sham acupuncture or rest‐only control condition was included. Therefore, the observed cerebral perfusion changes cannot be attributed specifically to acupuncture, and contributions from nonspecific somatosensory stimulation, contextual or expectancy‐related effects, and time‐related changes during repeated MRI acquisition cannot be excluded. Accordingly, the present findings should be interpreted as temporal cerebral perfusion changes associated with Japanese‐style acupuncture rather than as evidence of acupuncture‐specific effects. Future randomized neuroimaging studies incorporating both sham acupuncture and rest‐only control conditions will be necessary to distinguish acupuncture‐specific responses from nonspecific sensory, contextual, and time‐related effects.

Third, all six ASL scans were acquired in the same fixed temporal order within a single imaging session, from baseline through stimulation to 30 min after needle removal. Thus, the scan order was fully confounded with the elapsed time in the scanner. Although participants were instructed to remain awake with their eyes closed throughout the examination, vigilance and drowsiness were not objectively monitored. Therefore, time‐dependent physiological changes, scanner‐related drift, fatigue, or changes in vigilance may have contributed to the observed temporal perfusion patterns. In the absence of a rest‐only comparison condition, these potential time‐related effects cannot be separated from changes associated with acupuncture stimulation. Accordingly, the prolonged poststimulation perfusion patterns should not be interpreted as definitive evidence of a sustained acupuncture‐specific effect. Future studies incorporating appropriate time‐matched control conditions will be necessary to distinguish intervention‐related responses from temporal changes during repeated ASL acquisition.

Fourth, all MRI examinations were performed during the interictal period in patients with episodic migraine. Consequently, the present findings cannot be generalized to ictal migraine or chronic migraine, both of which may exhibit different patterns of cerebral perfusion and network organization.

Fifth, the present study focused on cerebral perfusion and did not directly evaluate clinical outcomes, autonomic function, neuropeptide concentrations, or functional connectivity. Future studies integrating ASL MRI with clinical outcome measures, HRV, molecular biomarkers such as CGRP, and multimodal neuroimaging techniques will provide a more comprehensive understanding of the neurophysiological mechanisms underlying Japanese‐style acupuncture.

In addition, the whole‐brain voxel‐wise analysis used an uncorrected statistical threshold (*p* < 0.01) with a minimum cluster extent of 100 voxels, without formal correction for multiple comparisons. This approach increases the risk of false‐positive findings, particularly given the small sample size. Therefore, the regional findings from the voxel‐wise analysis should be interpreted as exploratory and hypothesis‐generating rather than confirmatory. Future studies with larger samples and appropriately corrected whole‐brain analyses, such as FWE‐ or FDR‐corrected approaches, are required to confirm the spatial patterns observed in the present study.

Another limitation concerns the use of single‐delay PASL. ASL signal intensity can be influenced by arterial transit time (ATT), and ATT was not independently measured in the present study. Therefore, differences in ATT between patients with episodic migraine and healthy controls, as well as potential temporal changes in ATT during the experimental session, may have contributed to the observed ASL signal changes. Accordingly, the observed regional changes cannot be unequivocally interpreted as changes in tissue perfusion alone and may partly reflect transit time effects. Future studies using multidelay ASL or other approaches that permit estimation of ATT will be necessary to distinguish tissue perfusion changes from transit‐related effects.

Another important limitation concerns the use of proportional scaling to a fixed global mean of 50 mL/100 g/min. This procedure removes information regarding absolute global CBF and expresses regional perfusion values relative to the global mean. Consequently, apparent regional increases or decreases may partly reflect a relative redistribution of perfusion across brain regions rather than absolute changes in local tissue perfusion. In particular, the relatively lower occipital perfusion observed in patients with episodic migraine cannot be interpreted as evidence of an absolute reduction in occipital CBF and may partly reflect relative increases in other regions. Future studies retaining absolute perfusion information will be necessary to determine whether the regional patterns observed here correspond to true changes in local tissue perfusion.

Head motion represents another potential limitation of the serial ASL measurements. Because needle insertion and removal occurred during the imaging session, movement associated with these procedures or residual head motion between serial acquisitions may have influenced the spatial alignment and the observed perfusion patterns. Although participants with insufficient image quality were excluded from the final analysis, quantitative motion parameters were not available for evaluation in the present analysis. Therefore, motion‐related effects cannot be excluded as a potential contributor to the observed regional perfusion patterns. Future serial ASL studies should incorporate systematic motion assessment and report quantitative motion parameters across experimental phases and groups.

Finally, physiological variables that may influence cerebral perfusion were not continuously monitored during ASL acquisition, including end‐tidal CO_2_, respiration, heart rate, and blood pressure. Although participants were instructed to refrain from coffee, tea, and other caffeinated beverages before the MRI examination, caffeine intake was not objectively quantified. Therefore, residual physiological and caffeine‐related effects on cerebral perfusion cannot be completely excluded. Future studies should incorporate standardized physiological monitoring and more detailed control of caffeine exposure during serial ASL acquisition.

## 5. Conclusion

The present study identified temporally organized cerebral perfusion patterns during and after Japanese‐style acupuncture in patients with episodic migraine and healthy controls. Compared with healthy controls, patients with episodic migraine exhibited perfusion patterns that remained evident during the poststimulation period, suggesting differences in the temporal dynamics of cerebral perfusion between the two groups.

Repeated ASL MRI enabled the visualization of cerebral perfusion across multiple time points within a single experimental session. However, because the study did not include sham acupuncture or a rest‐only control condition and the serial scans were acquired in a fixed temporal order, the observed changes cannot be attributed specifically to acupuncture or interpreted as evidence of sustained acupuncture‐induced neuromodulation.

These findings should therefore be considered exploratory and hypothesis‐generating. Further studies incorporating appropriate control conditions, larger samples, and rigorous neuroimaging methodology are needed to clarify the physiological significance of these temporal cerebral perfusion patterns in episodic migraine.

## Funding

This study was supported in part by a Health and Labour Sciences Research Grant from the Ministry of Health, Labour and Welfare, Japan (Grant H24‐Iryo‐Ippan‐023) and by an institutional research grant from Saitama Medical University.

## Disclosure

All scientific content, analyses, and conclusions were developed and verified by the authors, who take full responsibility for the integrity and accuracy of this work.

## Conflicts of Interest

The authors declare no conflicts of interest.

## Data Availability

The data are not publicly available because of privacy and ethical restrictions. Requests for access to de‐identified demographic and clinical data may be directed to the corresponding author and will be considered on a case‐by‐case basis, subject to applicable ethical and institutional requirements. Access to MRI data requires additional consultation with the researcher responsible for those data and the relevant institution.
